# Novel B55α-PP2A mutations in AML promote AKT T308 phosphorylation and sensitivity to AKT inhibitor-induced growth arrest

**DOI:** 10.18632/oncotarget.11209

**Published:** 2016-08-11

**Authors:** Geoffrey Shouse, Rosalia de Necochea-Campion, Saied Mirshahidi, Xuan Liu, Chien-Shing Chen

**Affiliations:** ^1^ Division of Hematology/Oncology, Loma Linda University School of Medicine, Loma Linda, CA, USA; ^2^ Loma Linda University Cancer Center, Biospecimen Laboratory, Loma Linda University School of Medicine, Loma Linda, CA, USA; ^3^ Department of Biochemistry, University of California, Riverside, Riverside, CA, USA

**Keywords:** acute myeloid leukemia, AKT, protein kinase B, B55α, protein phosphatase 2A

## Abstract

Activation of the Protein Kinase B (PKB), or AKT pathway has been shown to correlate with acute myeloid leukemia (AML) prognosis. B55α-Protein Phosphatase 2A (PP2A) has been shown to dephosphorylate AKT at Thr-308 rendering it inactive. In fact, low expression of the PP2A regulatory subunit B55α was associated with activated phospho-AKT and correlated with inferior outcomes in AML. Despite this fact, no studies have specifically demonstrated a mechanism whereby B55α expression is regulated in AML. In this study, we demonstrate novel loss of function mutations in the PPP2R2A gene identified in leukemic blasts from three AML patients. These mutations eliminate B55α protein expression thereby allowing constitutive AKT activation. In addition, leukemic blasts with PPP2R2A gene mutation were more sensitive to treatment with the AKT inhibitor MK2206, but less responsive to the PP2A activator FTY720. Using leukemia cell lines, we further demonstrate that B55α expression correlates with AKT Thr-308 phosphorylation and predicts responsiveness to AKT inhibition and PP2A activation. Together our data illustrate the importance of the B55α-PP2A-AKT pathway in leukemogenesis. Screening for disruptions in this pathway at initial AML diagnosis may predict response to targeted therapies against AKT and PP2A.

## INTRODUCTION

Acute myeloid leukemia (AML) is a form of hematopoietic malignancy characterized by clonal expansion of proliferative, abnormally differentiated cells overwhelming the bone marrow leading to hematopoietic failure. The incidence of AML increases with age and it is the most common type of leukemia in adults. Despite its frequency, only modest advances have been made in the treatment outcome of this disease and these can be largely attributed to marked improvements in supportive care. Our knowledge and understanding of AML has been significantly advanced initially with identification of the importance of karyotype, and subsequently by molecular genetic mutations, expression profiles and whole genome sequencing [1]. The prognostic importance of such biologic heterogeneity in AML is well accepted and yet for over 3 decades the most widely accepted remission induction regimens include cytarabine and anthracyclines have not changed [2]. Although these regimens vary in dosing, the toxicities are significant and the treatment outcome worsens with age [3–6]. The interest is high in finding less toxic, more efficacious, novel molecular targeted therapies.

The complex cell signaling pathway involving the serine/threonine kinase AKT has been shown to correlate with AML prognosis [7]. AKT is an oncoprotein activated by phosphorylation at Thr-308 and Ser-473. Importantly, in a retrospective study, Thr-308 phosphorylation of AKT has been associated with high-risk cytogenetics and predicts poor overall survival in AML [8]. It has been shown previously that the regulatory subunit, B55α, is responsible for targeting the serine/threonine phosphatase PP2A to dephosphorylate AKT at Thr-308 leading to decreased AKT activity [9].

In fact, decreased B55α expression in AML is associated with increased levels of Thr-308 phospho-AKT and poor prognosis [10]. Despite this fact, the underlying mechanism regulating B55α expression in AML remains elusive.

In the present study, we identify two novel mutations in the PPP2R2A gene in leukemic blasts from three patients with de-novo AML. Both mutations lead to early termination codons and total loss of detectable B55α protein expression. The loss of B55α protein expression leads to loss of PP2A-AKT interaction and correlates with high levels of Thr-308 phospho-AKT and increased AKT activity. Finally, we demonstrate that the cells are significantly more sensitive to treatment with the AKT inhibitor MK2206. These findings demonstrate the importance of the B55α-PP2A-AKT pathway in AML. A subset of AML patients with disruptions in this pathway may be responsive to targeted therapy by AKT inhibition.

## RESULTS

### Identification of novel mutations in the B55α gene in AML blasts

**Figure 1:**
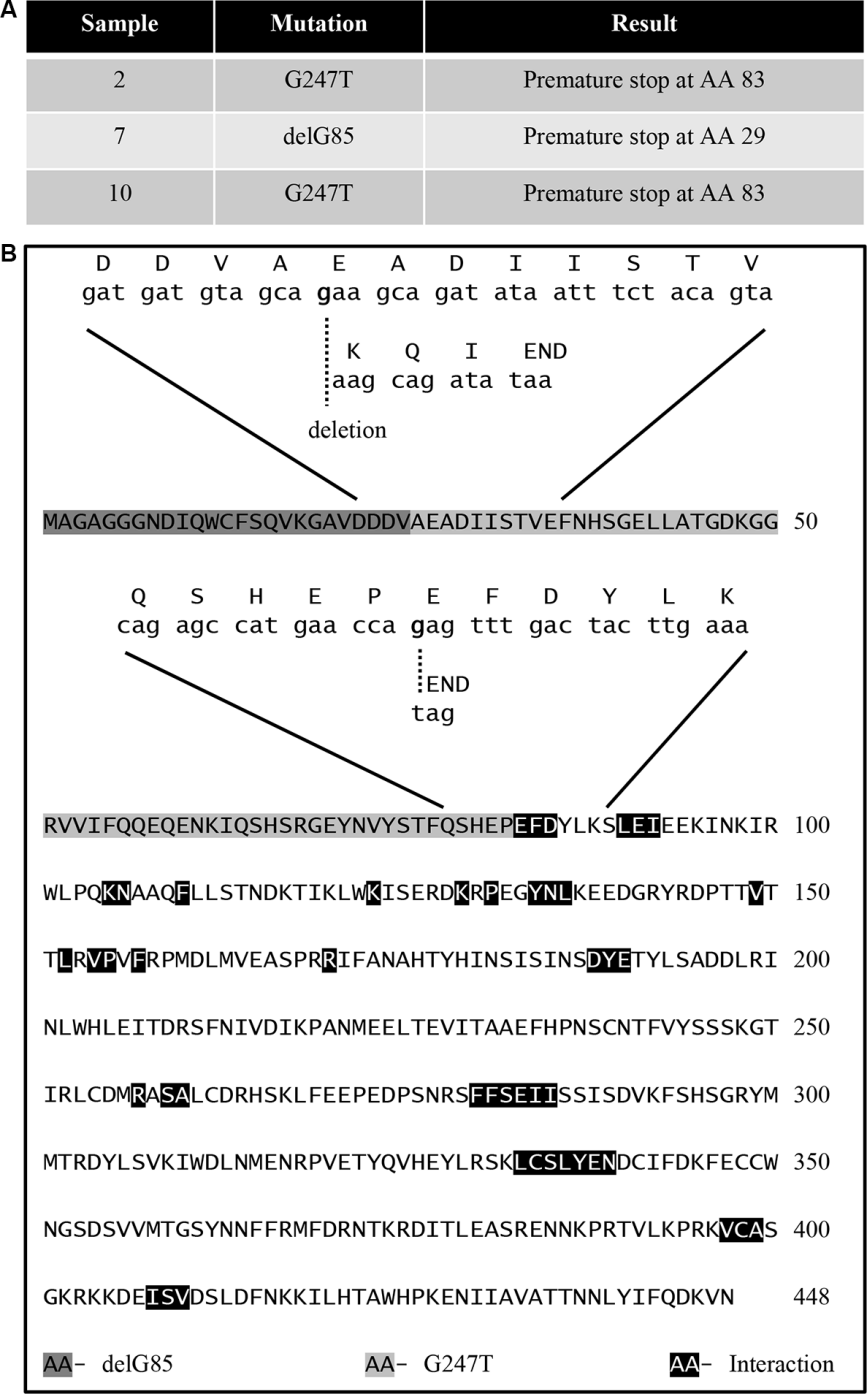
Identification of B55α mutations in AML blasts (**A**) Table showing the mutations identified in the indicated sample as well as the resultant change in amino acid sequence. (**B**) Amino acid sequence of B55α with superimposed aligned mRNA and amino acid sequences near the identified mutations also showing previously reported residues required for interaction between B55α and other proteins. AA: amino acid; Del: deletion; G: guanine; T: thymine.

A previous study showed that decreases in B55α protein expression in AML correlated with shorter complete remission duration. However, B55α mutations were not evaluated in this study. [10]. We evaluated the full length cDNA sequence of B55α in leukemic samples from 11 unselected patients with treatment naive, de novo AML. The clinical characteristics and patient identity remained unknown to the primary investigator until the sequencing results were completed. The purified B55α cDNA was sequenced and a novel transversion mutation, G247T was identified in samples 2 and 10, leading to a nonsense mutation at amino acid 83 of the B55α protein. In addition, in sample 7, the guanine residue at base pair 85 was deleted leading to a frame shift and generation of a premature stop codon at amino acid 29 (Figure 1A). The remaining 8 samples had the wild type sequence for B55α. Next, we sequenced exon 2 and 3 of the B55α gene and verified that the mutations were also present on the genomic DNA. Interestingly, the sequencing histogram demonstrated the presence of 2 allelic variants in each patient, the wild type and the mutant (Supplementary Figure S1). This suggests that the samples were heterozygous for the mutations and therefore the mutations were likely somatic in nature. More importantly, the cDNA sequences only identified the mutant alleles suggesting some underlying transcriptional regulatory mechanism is in place leading to preferential transcription of the mutant allele. We further analyzed the mutant B55α amino acid sequence against the published crystal structure of the B55α-PP2A complex that illustrated the residues important for interaction with the PP2A A and C subunits as well as for substrate binding [11]. Interestingly, the truncated mutant protein products lack all of the residues reportedly important for interaction with the PP2A A and C subunits as well as with the substrate (Figure 1B), suggesting that these mutations render the B55α protein non-functional based on the three-dimensional structural analysis. Analysis using predictive modeling also suggests that these mutations are deleterious to normal B55α protein function (Supplementary Figure S2) [12].

### B55α mutation correlates with loss of B55α expression, decreased PP2A activity and enhanced AKT phosphorylation in leukemic blasts

**Figure 2:**
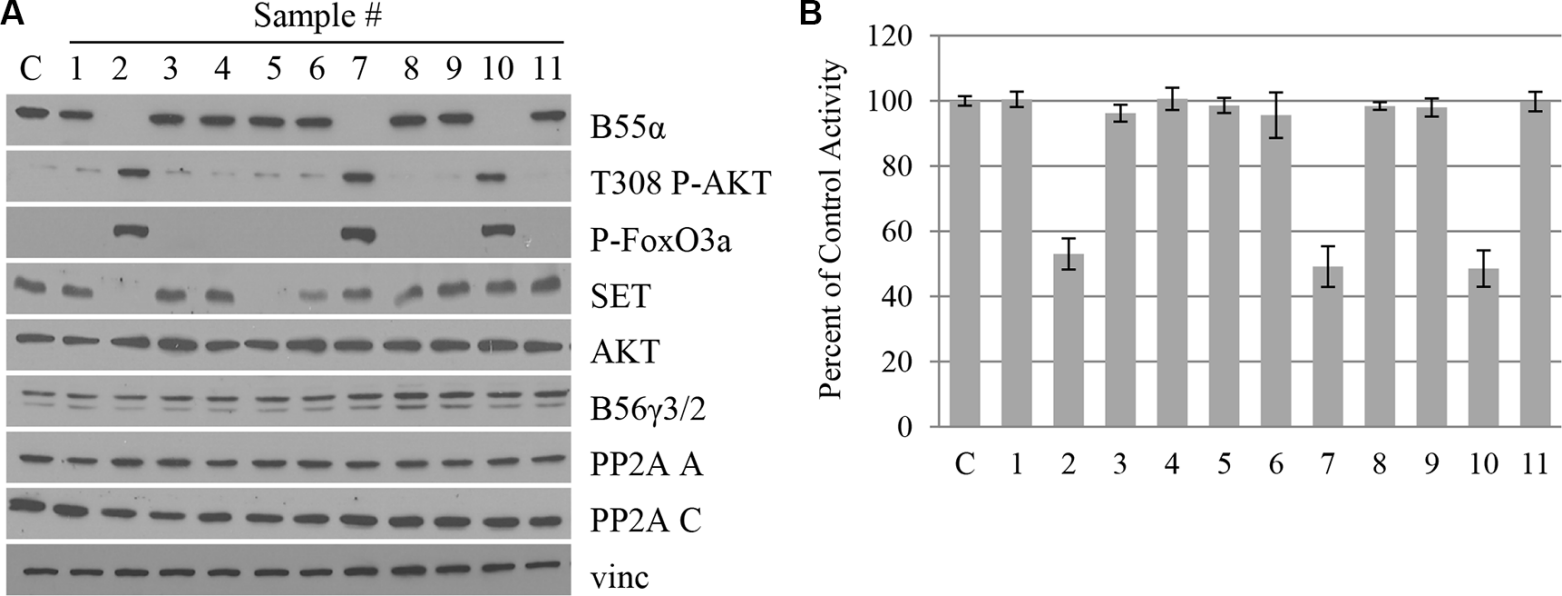
B55α mutation correlates with loss of B55α expression, decreased PP2A activity and enhanced AKT phosphorylation in leukemic blasts (**A**) Whole cell lysate from samples (1–11) or control cells (**C**) were subjected to western blotting with the given antibodies. (**B**) *In vitro* phosphatase assay was performed on cell lysates from samples (1–11) or control cells (C), and raw activity was compared to control and reported as a percentage. Bars represent average of triplicate experiments +/− standard deviation. P-FoxO3A: phosphorylated FoxO3A protein; Vinc: Vinculin.

B55α protein and mRNA are ubiquitously expressed in virtually all tissue types and reach a moderate level of expression in normal bone marrow [13]. Since the novel B55α mutations led to premature stop codons, we investigated the B55α protein expression levels in the primary leukemia patient samples (Figure 2A). As expected, B55α expression was eliminated in all 3 samples with mutations, but was present in each of the other 8 samples as well as a control lysate from an AML-derived cell line with intact B55α. Protein levels of the PP2A A and C subunits as well as another regulatory B subunit, B56γ, were similar in each of the samples. Interestingly, total AKT protein levels were unaffected by the B55α mutations. AKT Thr-308 phosphorylation, however, was significantly higher in the mutant samples. The AKT target, FoxO3A also showed increased levels of phosphorylation in the mutant samples. This finding supports the previously described notion that B55α is responsible for directing PP2A to dephosphorylate AKT at Thr-308 [9].

To further evaluate the effect of B55α mutation on PP2A activity, *in vitro* phosphatase assays were performed as previously described [14]. PP2A C subunit was immunoprecipitated and the phosphatase activity of the purified proteins was evaluated. The input for the immunoprecipitation is demonstrated in Figure 2A and the levels of immunoprecipitated C subunit are shown in Supplementary Figure S3. As shown in Figure 2B, samples 2, 7, and 10 with mutant B55α had a 50% reduction in the *in vitro* activity of PP2A. These findings suggest that loss of B55α function in these AML samples cripples the PP2A enzyme leading to elevated phosphorylation of the cellular kinase AKT. Interestingly, expression of SET, an endogenous protein inhibitor of PP2A [15], was variable in the different samples and did not seem to correlate with overall PP2A activity (Figure 2A).

### B55α mutations abolish PP2A-AKT interaction in leukemic blasts

**Figure 3:**
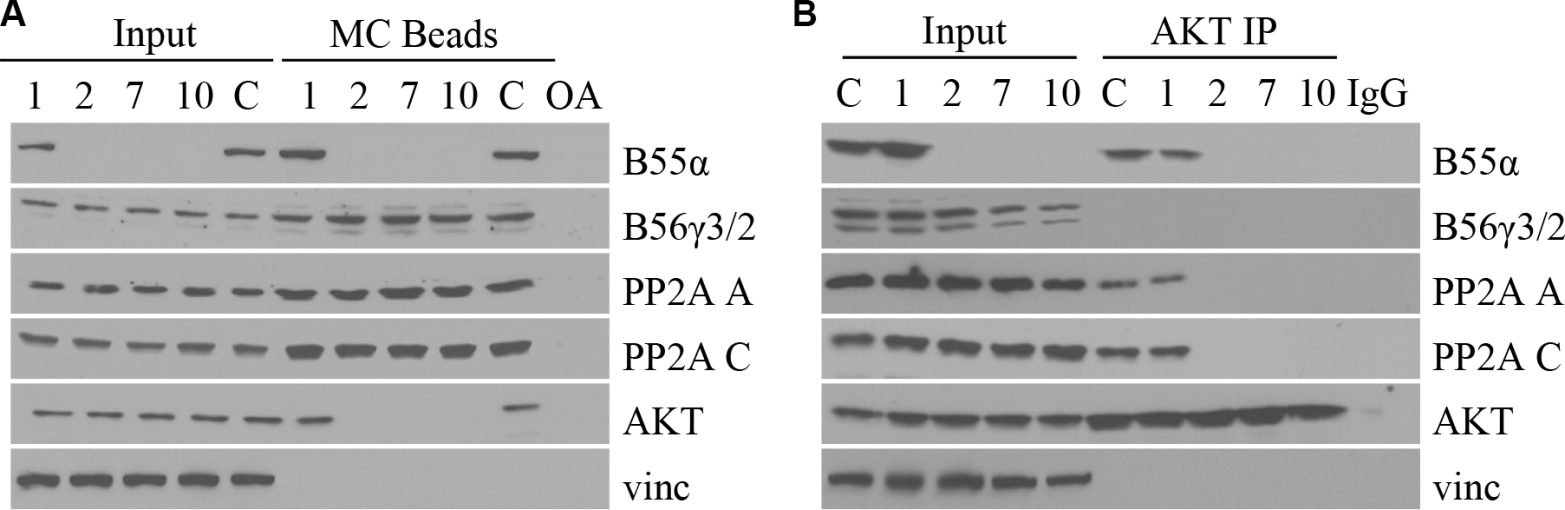
B55α mutations abolish PP2A-AKT interaction in leukemic blasts (**A**) Whole cell lysate from samples (1, 2, 7, 10) or control cells (**C**), were incubated with microcystin beads, washed then subjected to immunoblotting (MC Beads), along with 1% input (Input) with the antibodies listed. (**B**) Whole cell lysate from primary leukemia samples (1, 2, 7, 10) or control cells (C), were incubated with protein A agarose and AKT antibody, washed then subjected to immunoblotting (AKT IP), along with 1% input (Input) with the antibodies listed. IgG: immunoglobulin G negative control; OA: okadaic acid; Vinc: Vinculin.

Since the B55α mutations led to a decrease in PP2A activity as well as an increase in AKT phosphorylation, we investigated the effect of the B55α mutation on PP2A-AKT interaction. Samples were subjected to microcystin beads pull down, which precipitates the PP2A C subunit. Precipitated proteins were analyzed by Western Blotting. Figure 3A demonstrates that mutation in B55α, leads to loss of B55α interaction with the PP2A C subunit. More importantly, B55α mutation also led to loss of PP2A-AKT interaction. These findings provide further support to the notion that loss of B55α expression allows for constitutively active Thr-308 phospho-AKT to accumulate in leukemic blasts. As a control, another PP2A regulatory B subunit, B56γ was present in all lanes, suggesting B56γ-PP2A complexes are still formed normally when B55α is mutated. Reciprocally, using AKT immunoprecipitation, we found that AKT interaction with PP2A A and C subunits was detectable only when wild type B55α protein was present (Figure 3B). In the samples with B55α mutation, not only was AKT-B55α interaction lost, but AKT-PP2A interaction was lost as well. These findings reinforce the PP2A interaction studies discussed above and provide additional evidence for the molecular mechanisms disrupted by the B55α mutations present in these AML samples.

### B55α mutation predicts responsiveness to AKT inhibition and PP2A activation in leukemic blasts

**Figure 4:**
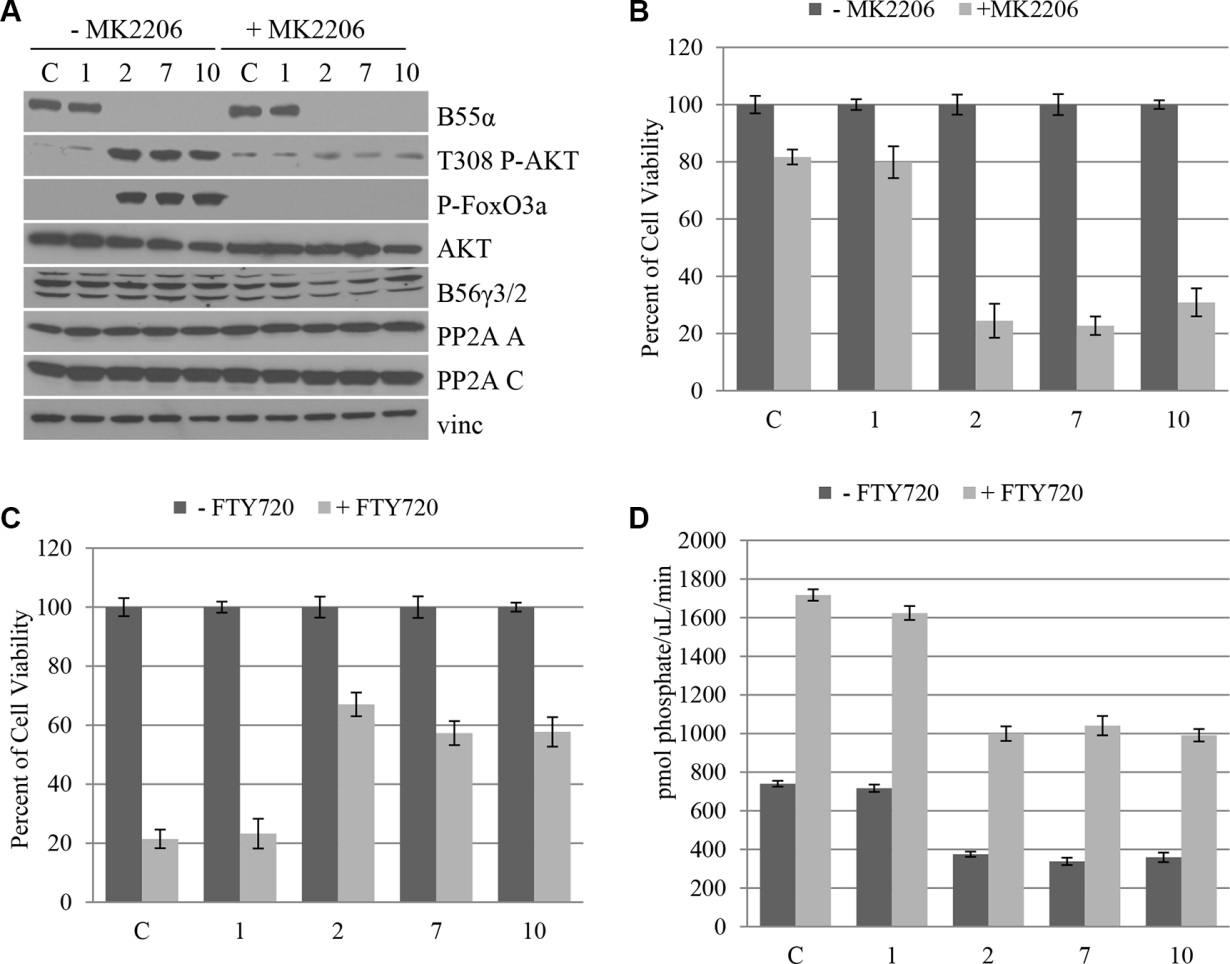
B55α mutation predicts responsiveness to AKT inhibition and PP2A activation in leukemic blasts (**A**) Cells from leukophoresis samples (1, 2, 7, 10) or control cells (**C**) were either mock treated (− MK2206) or treated with the AKT inhibitor MK2206 (+ MK2206), then lysed and subjected to western blotting with the indicated antibody. (**B**) Cells were either mock treated (− MK2206) or treated with MK2206 (+ MK2206) then subjected to MTT cell viability assay. Cell viabilities were reported as a percent of the mock treated viability. (**C**) Cells were either mock treated (− FTY720) or treated with FTY720 (+ FTY720) then subjected to MTT cell viability assay. Cell viabilities were reported as a percent of the mock treated viability. (**D**) *In vitro* phosphatase assay was performed on cell lysates from the leukophoresis samples (1, 2, 7, 10) or control cells (C) that were either mock treated (− FTY720) or treated with FTY720 (+ FTY720). Bars represent average of triplicate experiments +/− standard deviation. P-FoxO3A: phosphorylated FoxO3A protein; Vinc: Vinculin.

We demonstrated that B55α mutation leads to disruption of PP2A-AKT interaction as well as AKT activation. Based on this finding we investigated the effect of AKT inhibition using the chemical AKT inhibitor, MK2206, which is currently under investigation in clinical trials as an anticancer agent in solid tumors [16–22]. Cells from leukemic samples with either wild type (1), or mutant (2, 7, 10) B55α were treated with the AKT inhibitor MK2206 and subjected to Western Blotting (Figure 4A). The AKT Thr-308 phosphorylation and activation seen in the mutant samples were ablated by treatment with the inhibitor. Based on this finding, next we investigated the effect of AKT inhibition on cell viability (Figure 4B). The AML patient samples with B55α mutation were significantly more responsive to AKT inhibition. Nearly 80% of cell viability was lost after treatment with the AKT inhibitor in the mutant samples compared to only 20% in those that harbor wild type B55α, demonstrating that constitutive AKT activation is a key mechanism during leukemogenesis in these AML samples.

A small molecule activator of PP2A has previously been identified, namely fungolimod (FTY720) [23]. This molecule acts by disrupting interaction between the PP2A C subunit and the endogenous inhibitor protein SET [15, 24]. Previous studies have shown that the B55α subunit is upregulated in response to FTY720 treatment in AML patients with c-kit mutation [25] and cells with RNAi mediated knockdown of B55α expression are less responsive to FTY720 treatment [26] suggesting a role for B55α in tumor response to this potential chemotherapeutic. As such, we investigated the effect of this small molecule activator on leukemic blast cell viability. The sample with wild type B55α, sample 1, had a much more robust response to PP2A activation, with almost 80% decrease in cell viability, compared to only 40% decrease in the mutant samples (Figure 4C). This supports prior findings suggesting that AML with intact B55α expression responds well to FTY720 treatment, while AML with B55α mutation is less responsive to targeted PP2A therapy [10]. The Western Blot of cells treated with the PP2A activator demonstrate minimal effect of FTY720 on PP2A levels, AKT expression, AKT Thr-308 phosphorylation, or phosphorylation of the AKT target FoxO3A (Supplementary Figure S4). In addition, *in vitro* phosphatase activity of PP2A was enhanced in each treatment sample, however intact B55α protein expression led to higher absolute increases in PP2A activity compared to the mutant samples (Figure 4D). The levels of immunoprecipitated C subunit are shown in Supplementary Figure S5. Taken together our findings demonstrate novel mutations in the B55α gene in AML that lead to increased AKT phosphorylation and activity. More importantly, B55α mutation appears to sensitize leukemia cells to MK2206 but ablate response to FTY720 and may represent a potential biomarker for evaluating targeted therapy and individualized medicine in AML.

The status of the B55α gene appears to modulate leukemia cell response to both the AKT inhibitor MK2206 and the PP2A activator FTY720. The presence of intact B55α protein appears to be required for response to FTY720, while loss of this protein appears to sensitize cells to MK2206 treatment. Based on these findings we investigated the effect of treating leukemic blasts with both agents simultaneously (Supplementary Figure S6). The combination appeared to have an additive effect leading to up to 90% reduction in cell viability regardless of the presence of B55α gene mutations.

### B55α expression in leukemia cell lines correlates with AKT Thr-308 phosphorylation and predicts responsiveness to AKT inhibition and PP2A activation

**Figure 5:**
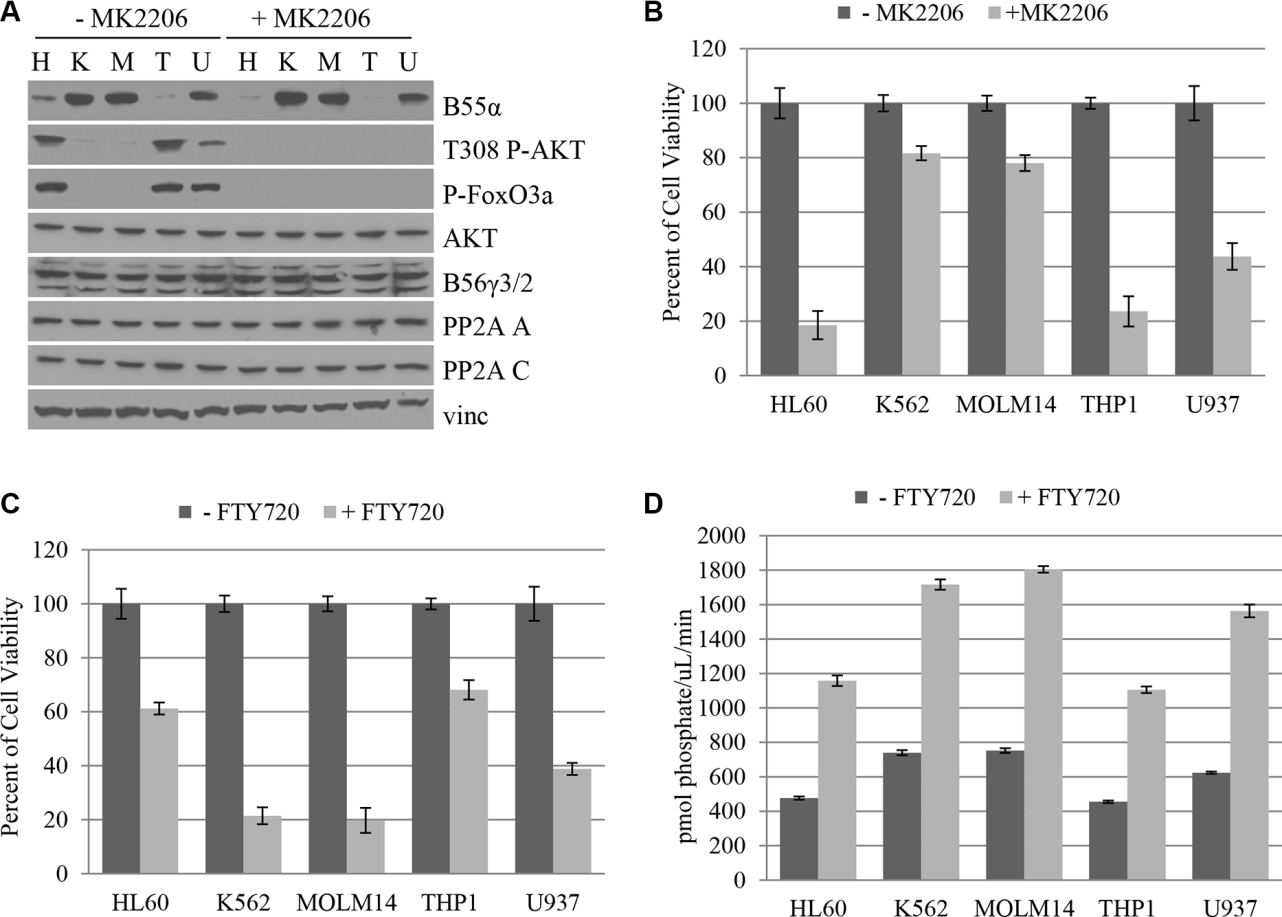
B55α expression in leukemia cell lines correlates with AKT Thr-308 phosphorylation and predicts responsiveness to AKT inhibition and PP2A activation (**A**) Cultured cells were either mock treated (− MK2206) or treated with the AKT inhibitor MK2206 (+ MK2206), then lysed and subjected to western blotting with the indicated antibody. (**B**) Cells were either mock treated (− MK2206) or treated with MK2206 (+ MK2206) then subjected to MTT cell viability assay. Cell viabilities were reported as a percent of the mock treated viability. (**C**) Cells were either mock treated (−FTY720) or treated with FTY720 (+ FTY720) then subjected to MTT cell viability assay. Cell viabilities were reported as a percent of the mock treated viability. (**D**) *In vitro* phosphatase assay was performed on cell lysates from the cell lines listed that were either mock treated (− FTY720) or treated with FTY720 (+ FTY720). Bars represent average of triplicate experiments +/− standard deviation. P-FoxO3A: phosphorylated FoxO3A protein; H: HL60; K: K562; M: MOLM14; T: THP1; U: U937; Vinc: Vinculin.

Since loss of B55α expression appears to be an important molecular disruption in AML, we investigated the cDNA and genomic DNA sequence as well as protein levels of B55α expression in five leukemia cell lines including HL60, K562, MOLM14, THP1 and U937. Interestingly, no mutations were detected in the B55α gene in these cell lines. Protein was purified from cultured cell lines and was subjected to immunoblotting. As shown in Figure 5A, HL60 and THP1 cells demonstrate low levels of B55α expression, while K562 and MOLM14 cells have high levels of expression. Finally, U937 had an intermediate level of B55α expression. Interestingly, levels of Thr-308 phosphorylation of AKT were inversely correlated with B55α protein expression, mimicking the pattern observed in primary leukemic blasts (Figure 2A). Levels of AKT activity showed a similar trend as shown by FoxO3A phosphorylation. AKT protein levels were similar in all cell lines as were levels of the PP2A A and C subunits and the B56γ regulatory subunit of PP2A.

We then investigated the effect of AKT inhibition using an AKT inhibitor, MK2206 (Figure 5B). The addition of MK2206 virtually abolishes the elevated AKT phosphorylation and activation seen in the HL60, THP1, and U937 cells, suggesting that this inhibitor is most effective in the setting of low levels of B55α expression. Since AKT inhibition had a clear effect on cell viability in the B55α mutant leukemia samples, we investigated its effect in leukemia cell lines. The HL60 and THP1 cell lines with the lowest initial levels of B55α expression had the most dramatic response to AKT inhibitor treatment at nearly 80% decrease in cell viability. On the other hand, the K562 and MOLM14 cells had only a modest decrease in cell viability of about 20%. U937 had an intermediate response of about 60% decrease in viability. The results demonstrate that the level of B55α expression inversely correlates with the responsiveness of the cells to AKT inhibitor treatment underscoring the importance of the B55α-PP2A-AKT pathway in promoting cell proliferation in cells with reduced B55α expression.

Next, we investigated the effect of PP2A activator treatment on the leukemia cell lines (Figure 5C). In the presence of FTY720, HL60 and THP1 cells, which lack robust B55α expression, showed only about 40% decrease in cell viability. The cell lines with higher B55α protein expression, K562 and MOLM14, had a much more dramatic response with as much as 80% decrease in cell viability. U937 had an intermediate response of 60% decrease in viability correlating with the intermediate B55α expression levels. AKT Thr-308 phosphorylation and AKT activity were not obviously affected by the treatment. Neither were protein levels of PP2A A, C, B55α, B56γ, or AKT (Supplementary Figure S7). The levels of immunoprecipitated C subunit are shown in Supplementary Figure S8. These findings suggest that B55α-PP2A may be one of the PP2A complexes activated after FTY720 treatment.

Since we previously showed that lack of B55α expression in AML leads to decreased PP2A activity (Figure 2B), we evaluated the level of PP2A activity in each of the leukemia cell lines (Figure 5D). The level of PP2A activity clearly correlated with B55α expression. Interestingly, all cell lines had significant increases in overall PP2A activity after FTY720 treatment. Despite this fact, B55α expression correlated with greater absolute increases of *in vitro* phosphatase activity as K562 and MOLM14 cells had the greatest overall PP2A activity after treatment, and HL60 and THP1 had the lowest activity. Taken together these findings demonstrate that B55α expression in leukemia cell lines correlates with sensitivity to two potential targeted therapeutics and provide further support to the results seen with the B55α mutant AML samples.

We further investigated the clinical and pathologic features of the three AML cases with mutations in PPP2R2A. The age of patients at diagnosis and initial presenting WBC were 30, 356,000 (86% blast); 65, 202,000 (98% blast); and 23, 26,000 (75% blast), respectively. All three were non-APL. One of the three cases was positive for NPM1 mutation only and the other was positive for both NPM-1 mutation and Flt-3-ITD. The immunophenotyping of blasts were positive for CD13, CD33, CD45, and CD117 in all three cases. Positive CD34 in concordance with HLA-DR was seen in two cases. The immunophenotype was non-specific as these markers were seen in patients not harboring PPP2R2A mutations (Supplementary Figure S9)

## DISCUSSION

The molecular genetic alterations in AML are increasingly being used to refine prognosis and are essential for target identification for potential therapeutic applications. In this study, for the first time, we identified two types of mutations in the PPP2R2A gene in leukemic blasts from three AML patients leading to loss of B55α protein expression and a significant increase in AKT Thr-308 phosphorylation. Of particular note, in our study, B55α expression was low in THP1 cells, however in a previous study it was reportedly high [26]. This discrepancy may be accounted for by differences in cell culture passage number, cell culture techniques and conditions, protein purification techniques, antibodies used for immunoblotting, or differences in relative expression compared to other cell lines. Regardless of this discrepancy, in the leukemia cell lines we tested, the level of B55α expression was inversely correlated with the level of AKT Thr-308 phosphorylation. Taking our findings together, we demonstrate a critical pathway in AML involving B55α-PP2A acting as a tumor suppressor by dephosphorylating AKT at Thr-308 and keeping it in an inactive form. In the case of our patient samples with PPP2R2A gene mutation, this tumor suppressive function of PP2A is lost and aberrant AKT signaling is present.

The importance of AKT in cancer has been studied and allosteric AKT inhibitors such as MK2206 have been studied in various early phase clinical trials [16–22, 27, 28] We verified the critical role for AKT in driving cell growth in the PPP2R2A mutant leukemic blasts by treating the cells with the AKT inhibitor, MK2206. We observed that the cell viability of the mutant samples was dramatically more sensitive to AKT inhibition than other samples or cell lines with intact B55α expression. Accordingly, in the leukemia cell lines tested, the level of B55α expression inversely correlated with sensitivity to MK2206. It follows that AML patients could be potentially screened at initial presentation for high levels of Thr-308 phospho-AKT using flow cytometry or ELISA as previously described [29]. Once an adequate system of screening tests are identified capable of distinguishing AML cases with perturbations in this pathway, a proof-of-principle trial could be designed for selected patients to receive a short course of AKT inhibitor prior to or concurrent with induction chemotherapy. Then the B55α-PP2A and phospho-AKT levels could be incorporated as correlative biomarkers assessed both pre and post targeted therapy. This approach would represent the next logical clinical development based on the findings elaborated in our study.

The importance of PP2A as a putative target in cancer therapeutics has led to identification of small molecule activators of PP2A phosphatase activity, including forksolin and fingolimod (FTY720) [30, 31]. FTY720 is in Phase III clinical trials due to its efficacy as treatment for multiple sclerosis as well as its action as an immunomodulator in renal transplant [32–37]. In addition, several preclinical studies suggest it is efficacious for specific leukemias as well, including blast crisis chronic myelogenous leukemia, Philadelphia chromosome positive ALL, and c-kit positive cancers, as well as other models for multiple myeloma, bladder and breast cancer, glioma, and hepatocellular carcinoma [23, 24, 35, 38–42]. In our study, PP2A activity was significantly diminished at baseline due to PPP2R2A mutation in leukemic blasts. Furthermore, PP2A activation in response to FTY720 was blunted in the mutant samples. This finding is in agreement with recent studies that showed B55α protein expression increased after FTY720 treatment [25] and B55α knockdown by RNAi in an AML cell line rendered the cells resistant to FTY720 [26]. This suggests a partial role for B55α-PP2A activity mediating the response to FTY720, although given the presence of response in leukemias lacking B55α expression, other functional PP2A enzymes that are targeted and activated cannot be excluded. This seems like a reasonable conclusion given that FTY720 appears to function by relieving the PP2A C subunit from its interaction with the endogenous inhibitor protein SET [15, 24]. In samples 7 and 10 that have detectable SET expression but lack B55α expression, however, the response to FTY720 was still blunted (Figure 2A, Figure 5A). Our findings can essentially differentiate a subgroup of AML bearing PPP2R2A gene mutation that may not respond to PP2A activation. Conversely, there is suggestion that AML with wild type [43–45] at normal expression levels may respond well to this type of treatment. Taken together our data underscore the potential benefit of screening AML patients for levels of B55α expression in a prospective therapeutic trial and AML patients with normal B55α expression could be selected for FTY720 therapy. Further evaluation of B55α antibodies may provide an effective screening tool with flow cytometry in the future.

Disruption of PP2A tumor suppressor function has been implicated in leukemogenesis in AML and other leukemias [46, 47]. Mechanisms include mutations in different PP2A subunit genes, changes in expression levels and post translational modifications of PP2A subunit proteins [43, 45, 48]. There have also been reports of PP2A inhibitory proteins including SET and SET Binding Protein 1, the over expression of which promotes leukemogenesis in AML by disrupting PP2A activity [43]. Additionally, B55α subunit expression levels have been previously shown to correlate with duration of remission in AML [10]. Although mutations in the PPP2R2A gene have been reported to occur as frequently as 15% in several types of solid tumors [48, 49], no prior studies have provided a conclusive mechanism for alterations in B55α expression levels in leukemia. Recently, there have been a plethora of attempts to identify mutations that promote either initiation or progression of AML using extensive targeted gene sequencing, expression profiling, or unbiased whole genome sequencing and no mutations in the PPP2R2A gene in AML have been reported [45, 50, 51]. To date more than 200 leukemia samples have been sequenced as part of the work on the Cancer Genome Atlas, yet no PPP2R2A gene mutation has yet been identified. This discrepancy may be explained by differences in techniques, differences in populations including differences in environmental exposure variables, or other factors not completely understood. The data we have suggest the patients harboring mutant PPP2R2A at least shared geographic similarity, even if their measured clinical variables did not show a clear pattern. However, it is well accepted that multiple genetic alterations lead to the development of AML and some relevant aberrations are yet to be discovered. In our study, the sequencing results suggest that the mutations we identified are heterozygous and most likely somatic. When we sequenced the genomic DNA, two alleles were found, the wild type and the mutant allele (Supplementary Figure S1). However, when we sequenced the cDNA, which was generated by RTPCR from purified mRNA, we only found the mutant allele. Essentially the mRNA of the wild type allele was undetectable. This suggests that although a wild type allele is present in the genomic DNA, it is not expressed. The lack of expression may be due to regulation of transcription, regulation of the stability of the mutant or wild type mRNA, or other process Unfortunately, normal matched tissue samples are not available for additional testing to answer whether the gene mutations were acquired or inherited, however given the fact that lack of B55α expression causes embryonic lethality, the former seems more likely [52]. Although we have identified one mechanism of regulation of B55α expression in AML, there are likely many other mechanisms in place in AML that lead to activation of AKT. PP2A is a complicated molecule with dozens of subunit genes leading to a plethora of functional enzymes with different subunit compositions. The regulation of expression of each of the subunits is also highly regulated at the level of transcription, translation, mRNA stability, variation in splice isoforms, protein stability, post translational modifications including phosphorylation, ubiquitination, and other mechanisms. In addition, the relative expression of particular B subunits can influence the interaction between the PP2A core and other subunits, meaning that if one B subunit is expressed at high enough levels it can replace other B subunits in the heterotrimeric complex. Without the AC core bound, many B subunits become unstable and are rapidly degraded. In addition to the inactivating PPP2R2A mutations discussed in this study, any of these other mechanisms and likely multiple of them are responsible for the decreased B55α expression seen in leukemic blasts in our study and others.

Although in our particular study, we investigated a limited number of unselected samples, additional collaborative work in more than fifty additional pediatric AML cases did not identify any additional B55α mutations, suggesting that mutations in this gene in AML is either a rare occurrence, or not common in childhood AML. We are continuing to collect additional adult AML samples to evaluate the frequency of this mutation on a larger scale. Despite this fact, we elucidated the underlying cellular pathway that is disrupted by these mutations, underscoring the importance of our findings.

## MATERIALS AND METHODS

### Ethics statement

Investigation has been conducted in accordance with the ethical standards and according to the Declaration of Helsinki and according to national and international guidelines and has been approved in accordance with the Loma Linda University Cancer Center Biospecimen Laboratory, with associated protocols approved by the Institutional Review Board of Loma Linda University. Written informed consent was obtained for the use of the samples in research.

### PCR primer design, RT-PCR, and sequencing

Total cellular RNA was purified from leukemic blasts using the Thermo Scientific (Waltham, MA) GeneJET RNA Purification kit. mRNA was then subjected to RT-PCR with oligo dT primers to amplify total mRNA, using the GoScript RT-PCR system from Promega (Madison, WI). cDNA was then purified using the Thermo Scientific GeneJET Gel Extraction and DNA Cleanup Micro Kit. PCR primers were designed against the 3′UTR of the published sequence of the B55α mRNA (PPP2R2A; 5′-GCAACATGGCAGGAGCTG-3′) as well as an internal primer (5′-CCACAATGTTAAAACTCCTGTCTG-3′), its reverse complement (5′-CAGACAGGAGTTTTAACATT GTGG-3′) and a 5′UTR primer (5′-GGAATGCCAACC CTAATTCAC-3′). Using this methodology, full length B55α cDNA was amplified as two fragments. PP2A A subunit was amplified as a control (PPP2R1A; 5′-GGAGCCAAGATGGCGGC-3′, 5′-CGCACCGAGTC CTGCTCG-3′). Beta Actin was amplified as a loading control (5′-GGGACGACATGGAGAAAA-3′, 5′-AAGG AAGGCTGGAAGAGTGC-3′). PCR product size was verified by agarose gel electrophoresis. Once the mutations were identified, additional primers were designed to amplify the genomic DNA of B55α exon 2 (5′-GGTATGTTTTCTTTTTCCAGGAGC-3′; 5′-CCTC CAGTAGGAAACATTTCTGC-3′) and 3 (5′-GAAA TATTTTTCAACAATGGTCCATA-3′; 5′-GGGCACAT GGAAAGAAAATATG-3′) to verify the presence of the mutations. Genomic DNA was purified using the GeneJET Gel Extraction and DNA Cleanup Micro Kit. The genomic DNA was amplified by PCR using the Thermo Scientific Maxima Hot Start PCR kit and the B55α primers. The PCR products were evaluated by agarose gel electrophoresis. All PCR primers were synthesized by MWG Operon (Huntsville, AL). Purified PCR product was shipped to Eton Biosciences (San Diego, CA) for sequencing.

### Cell culture and cell viability

Primary leukemia samples were obtained from routine diagnostic assessment or therapeutic leukapheresis on patients with treatment naive, de novo AML, presenting with symptomatic hyperleukocytosis. Low passage cells of the HL60, K562, THP1, and U937 were obtained from the American Type Culture Collection, while MOLM14 cells were obtained from the German Collection of Microorganisms and Cell Culture (Leibniz Institute DSMZ, Germany) and were defrosted and cultured in RPMI medium supplemented with 10% Fetal Bovine Serum, 2 mM L-glutamine, 100 U/ml penicillin and 100 μg/ml streptomycin. All cells were cultured in a 37°C humidified incubator and an atmosphere of 5% CO_2_ in air. After either mock treatment or treatment with MK2206 or FTY720, the cell viability was estimated using the Promega Cell Titer 96 Non-Radioactive Cell Proliferation Assay, an MTT (3-[4,5-Dimethylthythiazol-2-yl]-2,5-Diphenyltetrazolium Bromide) based assay, as previously described [21].

### AKT inhibitor, PP2A activator and phosphatase activity

The AKT inhibitor MK2206 and the PP2A Activator FTY720, were both obtained from Santa Cruz Biotechnology (Dallas, TX). Actively dividing cultured cells were treated either with the AKT inhibitor MK2206 [21] or the PP2A Activator FTY720 [14, 23] or the combination of both, as previously described. Cells were treated with 5 μM concentration of MK2206 or mock treatment and harvested after 24 hours. In separate experiments cells were treated with 2.5 μM concentration of FTY720 or mock treatment and harvested after 36 hours. When the two molecules were combined, the same concentrations were used, and both FTY720 and MK2206 were added simultaneously and harvested after 30 hours. PP2A phosphatase activity was estimated using the PP2A immunoprecipitation phosphatase assay kit from Millipore (Billerica, MA), as previously described [14] using antibodies to immunoprecipitate the PP2A C subunit. Protein level controls for western blotting and phosphatase assays were taken from lysed K562 cells as they had detectable B55α protein levels and low levels of background phosphorylated AKT (Figure 2A).

### Western blot and immunoprecipitation

Protein was purified from cells in culture and samples using the Thermo Scientific protein purification kit. Western Blotting was performed using antibodies against AKT (C-20, Santa Cruz Biotechnology), Phospho-AKT Ser-473 (Santa Cruz Biotechnology), Phospho-AKT Thr-308 (Santa Cruz Biotechnology), Phospho-FoxO1 (Thr24)/FoxO3A (Thr32) (Cell Signaling), PP2A A (Upstate), PP2A C (1D6, Upstate; polyclonal, Life Technologies), PP2A B55α (Upstate), PP2A B56γ [53], SET (H-120, Santa Cruz Biotechnology), and vinculin (VIN-11-5, Sigma). Immunoprecipitation was performed using protein A agarose and incubating with either AKT antibody or IgG as a negative control. Microcystin binding assay was performed using microcystin agarose beads (Upstate). For a negative control, lysates were incubated with 1.0 μM okadaic acid, a potent inhibitor and binder of the PP2A C subunit that interferes with binding to the microcystin beads.

## SUPPLEMENTARY MATERIALS FIGURES


