# Supplementary material for: Novel B55α-PP2A mutations in AML promote AKT T308 phosphorylation and sensitivity to AKT inhibitor-induced growth arrest
Source: Oncotarget. 2016 Aug 11;7(38):61081–92. doi: 10.18632/oncotarget.11209 (PMC5308637; doi:10.18632/oncotarget.11209)
Supplement: Supplementary file 1 [file oncotarget-07-61081-s001.pdf]

Novel B55α-PP2A mutations in AML promote AKT T308 phosphorylation and sensitivity to AKT inhibitor-induced growth arrest

Supplementary Materials

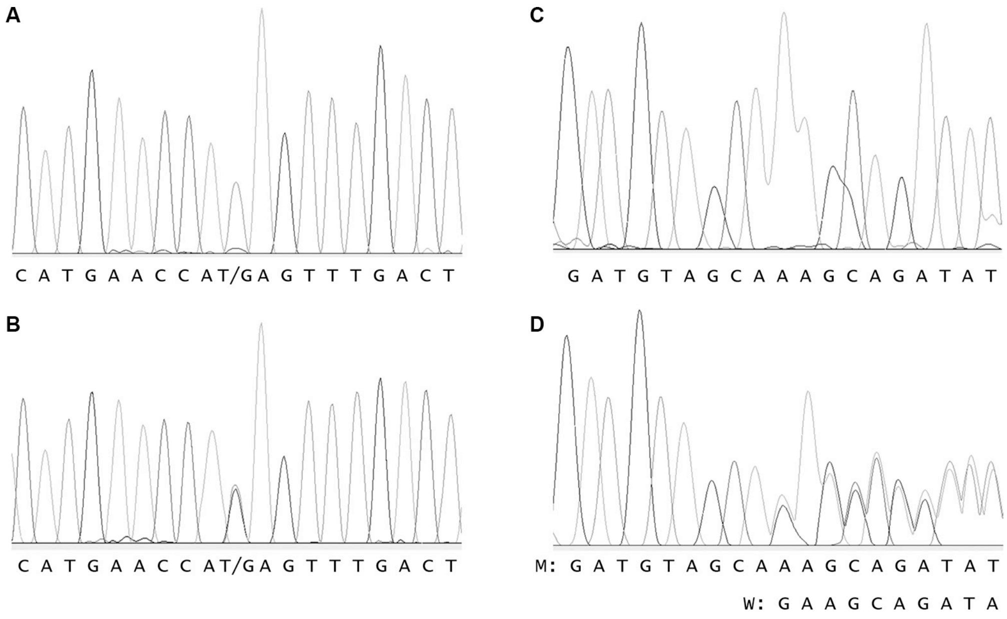

Supplementary Figure S1: Sequencing histograms from samples of AML blasts. Demonstration of sequencing histograms from sample 2 mRNA (**A**), genomic DNA (**B**), or sample 7 mRNA (**C**), or genomic DNA (**D**). A: adenine; C: cytosine; G: guanine; T: thymine; M: mutant allele sequence; W: wild type allele sequence.

Sample	Score	Predicted Result	
2	1	Deleterious	All known protein motifs lost
7	0.99	Deleterious	All known protein motifs lost
10	1	Deleterious	All known protein motifs lost

Supplementary Figure S2: Results from mutation prediction testing. Table showing the predicted results of the mutations on B55α function.

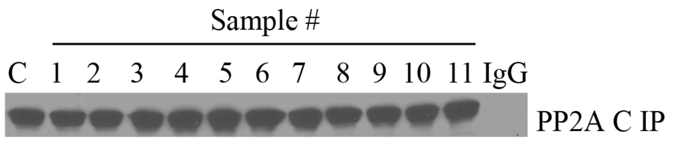

Supplementary Figure S3: PP2A C subunit IP input for PP2A activity assay. Western blot showing the amount of PP2A C subunit immunoprecipitated in the *in vitro* PP2A phosphatase activity assay shown in Figure 2B. 2C: control cells.

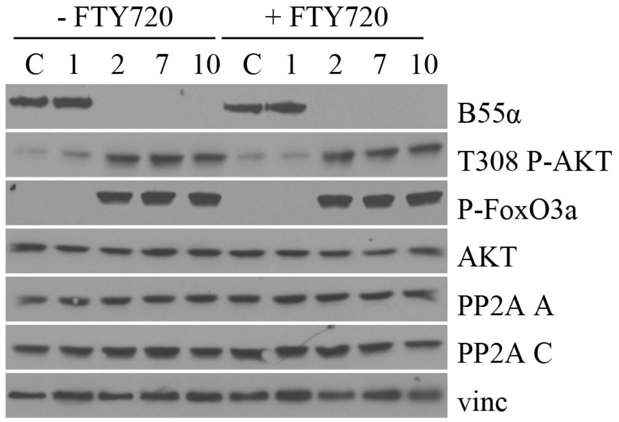

Supplementary Figure S4: Effect of PP2A activator on leukemic blasts. Cells were either mock treated (– FTY720) or treated with the PP2A activator FTY720 (+ FTY720), then lysed and subjected to western blotting with the indicated antibody. C: control cells; P-FoxO3A: phosphorylated FoxO3A protein; Vinc: Vinculin.

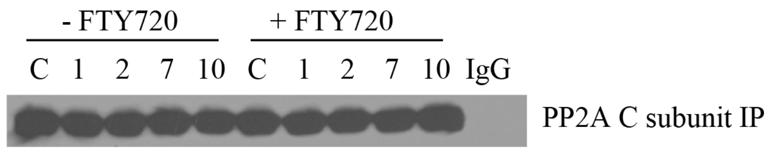

Supplementary Figure S5: PP2A C subunit IP input for activity assay. Western blot showing the amount of PP2A C subunit immunoprecipitated in the *in vitro* PP2A phosphatase activity assay shown in Figure 4D.

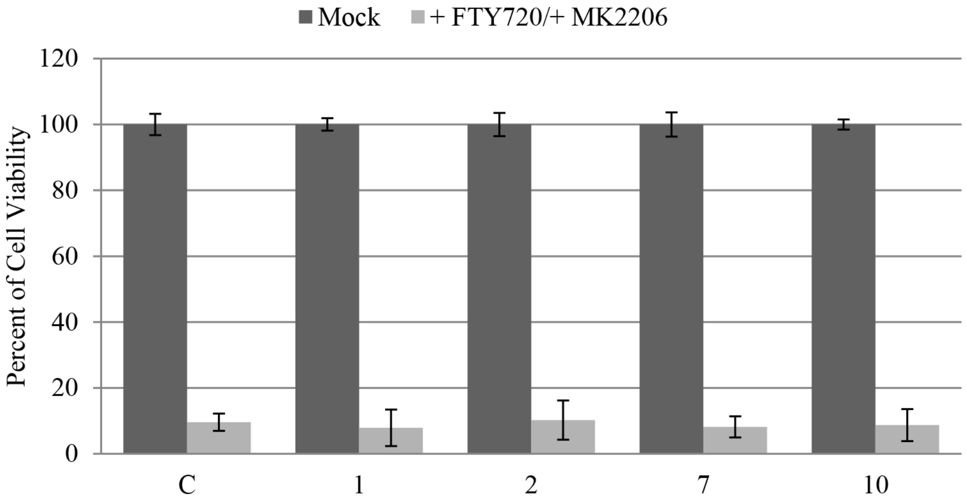

Supplementary Figure S6: Effect of treatment of leukemic blasts with MK2206 and FTY720. Cells were either mock treated (mock) or treated simultaneously with FTY720 and MK2206 (+ FTY720/+ MK2206) then subjected to MTT cell viability assay. Cell viabilities were reported as a percent of the mock treated viability. Bars represent average of triplicate experiments +/– standard deviation.

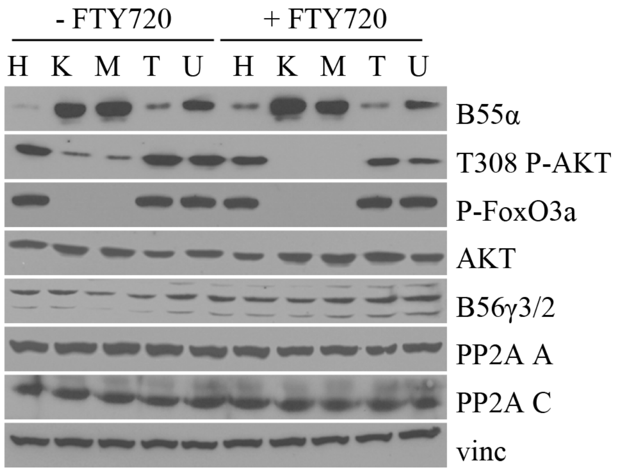

Supplementary Figure S7: Effect of PP2A activator on leukemia cell lines. Cultured cells were either mock treated (− FTY720) or treated with the PP2A activator FTY720 (+ FTY720), then lysed and subjected to western blotting with the indicated antibody. P-FoxO3A: phosphorylated FoxO3A protein; H: HL60; K: K562; M: MOLM14; T: THP1; U: U937; Vinc: Vinculin.

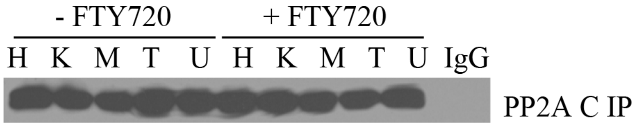

Supplementary Figure S8: PP2A C subunit IP input for PP2A activity assay. Western blot showing the amount of PP2A C subunit immunoprecipitated in the *in vitro* PP2A phosphatase activity assay shown in Figure 5D. H: HL60; K: K562; M: MOLM14; T: THP1; U: U937.

Sample #	Age	Sex	Race	AML Type	Blast Count	Immunophenotype	Mutation
1	59	M	Caucasian	M5a	183.78 K	cd2, cd11b, cd11c, cd13, cd33, cd36, cd38, cd45, cd56, cd64	3 copies of RARA
2	23	F	Caucasian	M1	26.78 K	cd7, cd13, cd33, cd34, cd45, cd 71, cd117, hla-dr, mpo	FLT3 ITD, NPM1
3	92	F	Caucasian	MDS -> M1	89.6 K	unknown	unknown
4	66	M	Hispanic	M1	137.27 K	cd7, cd13, cd33, cd38, cd43, cd45, cd117, hla-dr	FLT3 ITD, NPM1
5	56	F	Caucasian	M3	102.47 K	cd9, cd13, cd33, cd34, mpo	negative
6	51	F	Hispanic	M5b	82.07 K	NSE, sudan black, MPO	unknown
7	65	M	Caucasian	NOS	202.0 K	cd7, cd13, cd33, cd34, cd45, cd71, cd117, HLA-DR	NPM1
8	45	F	Caucasian	M0 vs M2	113.65 K	cd7, cd9, cd13, cd34, cd38, cd45, cd117, HLA-DR	unknown
9	34	M	Hispanic	M0 vs M1	29.36 K	cd13, cd33, cd34, cd38, cd45, cd117, HLA-DR, TDT	negative
10	30	F	Hispanic	M1	102.85 K	cd7, cd13, cd33, cd34, cd45, cd71, cd117, HLA-DR, MPO	FLT3 ITD, NPM1
11	61	F	African American	NOS	219.49 K	unknown	unknown

Supplementary Figure S9: Table of patient data. Blast counts are in cells per micro liter. F: Female; K: thousand; M: Male.
